# “I want to get better, but…”: identifying the perceptions and experiences of people who inject drugs with respect to evolving hepatitis C virus treatments

**DOI:** 10.1186/s12939-021-01420-7

**Published:** 2021-03-19

**Authors:** Trevor Goodyear, Helen Brown, Annette J. Browne, Peter Hoong, Lianping Ti, Rod Knight

**Affiliations:** 1 School of Nursing, University of British Columbia, Vancouver, Canada; 2 British Columbia Centre on Substance Use, 400-1045 Howe St, V6Z 2A9 Vancouver, BC Canada; 3 Department of Medicine, University of British Columbia, Vancouver, Canada

**Keywords:** Hepatitis C, Direct‐acting antivirals, Treatment, People who inject drugs, Substance use, Harm reduction, Qualitative research, Equity, Ethics, Health services

## Abstract

**Background:**

The advent of highly tolerable and efficacious direct-acting antiviral (DAA) medications has transformed the hepatitis C virus (HCV) treatment landscape. Yet, people who inject drugs (PWID) – a population with inequitably high rates of HCV and who face significant socio-structural barriers to healthcare access – continue to have disproportionately low rates of DAA uptake. The objective of this study is to explore how PWID with lived experience of HCV perceive and experience DAA treatment, in a setting with universal coverage of these medications since 2018.

**Methods:**

Informed by a critical interpretive framework, we thematically analyze data from in-depth, semi-structured interviews conducted between January and June 2018 in Vancouver, Canada, with a purposive sample (*n* = 56) of PWID at various stages (e.g., pre, peri, post) of DAA treatment.

**Results:**

The analysis yielded three key themes: (i) life with HCV, (ii) experiences with and perceptions of evolving HCV treatments, and (iii) substance use and the uptake of DAA treatments. First, participants described how health and healthcare conditions, such as the deprioritizing of HCV (e.g., due to: being asymptomatic, healthcare provider inaction, gatekeeping) and catalysts to care (e.g., symptom onset, treatment for co-morbidities) shaped DAA treatment motivation and access. Second, participants described how individual and community-level accounts of evolving HCV treatments, including skepticism following negative experiences with Interferon-based treatment and uncertainty regarding treatment eligibility, negatively influenced willingness and opportunities to access DAAs. Concurrently, participants described how peer and community endorsement of DAAs was positively associated with treatment uptake. Third, participants favoured HCV care that was grounded in harm reduction, which included the integration of DAAs with other substance use-related services (e.g., opioid agonist therapy, HIV care), and which was often contrasted against abstinence-focused care wherein substance use is framed as a contraindication to HCV treatment access.

**Conclusions:**

These findings underscore several equity-oriented healthcare service delivery and clinician adaptations that are required to scale up DAAs among PWID living with HCV, including the provision of harm reduction-focused, non-stigmatizing, integrated, and peer-led care that responds to power differentials.

## Introduction

Hepatitis C virus (HCV) infection remains a major contributor to worldwide morbidity and mortality [1]. While potentially curative HCV treatments have been available for nearly two decades, the previous and longstanding generation of Interferon-based treatments demonstrated limited effectiveness (~ 50 % cured), required prolonged treatment for up to 48 weeks, and had significant side effects (e.g., flu-like symptoms, insomnia, impaired mood) [2]. As such, rates of Interferon-based treatment uptake have been as low as 15 % among people living with HCV [3], and even lower among populations who are most marginalized with respect to treatment access, including people who inject drugs (PWID) [4, 5]. The recent advent of all-oral direct-acting antiviral (DAA) HCV treatments has renewed optimism for transforming the global HCV care landscape. These novel treatments have minimal side effects and contraindications and are highly effective in achieving HCV cure [6, 7]. Accordingly, the scale-up of DAAs constitutes a critical component underlying the World Health Organization’s goal to eliminate HCV as a major public health threat by 2030 [8].

Public health efforts to reduce the global burden of HCV must be firmly grounded in equity and social justice. Already, there have been calls to prioritize DAA treatment scale-up efforts with populations who face inequities in HCV prevalence, incidence, and healthcare access [9, 10]. PWID have been widely recognized as one such “priority population” – particularly, in regions wherein HCV rates among this population remain high [10–13]. Due to the sharing of injection drug equipment and the lack of accessible harm reduction services (e.g., needle and syringe distribution programs, opioid agonist therapy [OAT]) in various global settings, PWID continue to be inequitably impacted by blood-borne infections, including HCV and human immunodeficiency virus (HIV) [14, 15]. Indeed, PWID are estimated to comprise 6.1 million (8.5 %) of the 71.1 million prevalent chronic HCV cases worldwide [11, 16]. And in many ‘western’ countries, including Canada, where, as of 2015, approximately 53 % of PWID were estimated to be living with HCV [11], injection drug use is now the principal route of incident HCV transmission [8, 17].

While Interferon-free HCV therapies represent a “game changer” for PWID living with HCV, a series of barriers to equitable DAA treatment access remain. For instance, recent studies have described key implementation challenges related to specialist-centered models of HCV care, lack of on-site phlebotomy services, finite resources to develop a comprehensive HCV cascade of care, and onerous diagnostic and prescription-related eligibility requirements for accessing DAAs [18–20]. A small but growing body of empirical evidence with PWID participants also has identified the ways in which individual and socio-contextual factors influence DAA treatment access and uptake. For example, previous qualitative studies – few of which are from the current DAA treatment era – have examined how perceptions and knowledge levels related to various aspects of HCV and its management (e.g., disease transmission and progression, medication side effects, treatment eligibility and conditions, potential re-infection) are important determinants of HCV treatment uptake [21–24]. More generally, disinclination to undergo DAA treatment among PWID can stem from being asymptomatic of HCV, the prioritization of other health and social issues (e.g., co-morbidities, housing, childcare), and experiences of stigma and discrimination when accessing services [21, 25, 26]. Of particular concern, available evidence indicates that healthcare providers tend to view active substance use as a contraindication to DAA treatment eligibility, including due to a presumed lack of stability and/or capacity for PWID to adhere to treatment regimens, and the anticipated potential for sustained liver damage and/or re-infection following HCV cure [27–29].

Taken as a whole, PWID continue to face a set of interconnected challenges that restrict access to HCV care, including DAA therapies. As a consequence of these healthcare access inequities, PWID populations experience low rates of DAA treatment uptake, despite paradoxically experiencing both a high prevalence of HCV and a well-documented willingness to undergo treatment [8, 15, 30]. To address this noteworthy gap in equitable health and healthcare access among PWID living with HCV, there is a need for knowledge about how PWID’s experiences are unfolding within and influenced by evolving HCV care landscapes. The objective of this study is therefore to explore how PWID with lived experience of HCV perceive and experience DAA treatments, in a setting with universal coverage of these medications since 2018.

## Methods

### Theoretical perspectives

This study is informed by selected critical theoretical perspectives on health equity and social justice [31–33]. The first of these theoretical perspectives, as described in detail by Browne & Reimer-Kirkham [31], relates to dialectical social justice and provides a scaffolding for social justice scholarship to include both deconstructive (i.e., critical of oppression) and reconstructive (i.e., emancipatory) aims and analytical processes. The second theoretical perspective, by Varcoe and colleagues [32], outlines an equity-transformative framework for further bridging gaps between critical scholarship and practice (i.e., between knowledge and action). Building on these conceptualizations of social justice and health equity, we approach this study with a view toward advancing equity-oriented healthcare [33] with PWID who have lived experience of HCV. Described in depth elsewhere [33], equity-oriented healthcare and research is fundamentally concerned with addressing: “the effects of structural inequities (such as poverty), including the inequitable distribution of the determinants of health (e.g., income and housing) that sustain health inequities; the impact of multiple and intersecting forms of racism, discrimination and stigma [. .] on people’s access to services and their experiences of care; and the frequent mismatches between dominant approaches to care [. .] and the needs of people who are most affected by health and social inequities.” Within the current study, we operationalize these theoretical perspectives [31–33] to interrogate how HCV-related treatment experiences are embedded within the socio-structural conditions of PWID’s lives, and also to direct our analytical gaze toward the distinct contexts in which equitable access to DAAs is (or is not) realized for PWID with lived experience of HCV.

We also approach this study within a critical interpretive framework informed by our direct clinical and research experiences working with communities of PWID, including as public health researchers and as nurses. Here, we employ a thematic analysis approach that is grounded in a social constructivist epistemology [34, 35]. We operationalize this methodology by drawing on participants’ lived experiences and our interpretations as analysts, and by inductively approaching data analysis in a way that is firmly grounded in our theoretical and axiological positioning as critical health researchers. Through this lens, we interpret the lived experiences of HCV care among PWID to identify strengths-based and contextually-informed strategies for promoting equity-oriented healthcare with this population, particularly in the context of HCV.

### Study setting

This research was conducted in Metro Vancouver, British Columbia (BC), Canada, a metropolitan area with an approximate population of 2,463,431 people [36]. In Canada, the majority of healthcare services, including those under the umbrella of HCV care, are publicly funded and universally offered. Importantly, however, Canada does not have a federal pharma-care program, so publicly funded medication coverage, when available, is determined at the provincial level. In 2018, the province of BC removed restrictions from the BC PharmaCare program to DAA access and approved the universal coverage of several DAA treatment formulations for all British Columbians living with HCV [37]. This policy change expanded treatment access to the 53,441 HCV-diagnosed-individuals living in BC in 2018, among whom 18,609 (34.8 %) reported current or past injection drug use [38]. However, also in 2018, only 5,200 (27.9 %) individuals within this population of PWID living with HCV were able to access and begin treatment (with either DAAs or Interferon-based therapies), signaling an ongoing gap in the HCV cascade of care for this priority population [38].

Given the colonial context of Canada, it is important to understand the structurally-embedded nature of the inequities experienced by Indigenous^1^ PWID living with HCV, broadly, as well as the overrepresentation of Indigenous Peoples within the current study, specifically. Across Canada, Indigenous Peoples are inequitably impacted by substance use and HCV. As described in detail elsewhere (e.g., [26, 39, 40]), colonial policies and institutions in Canada (e.g., the Indian Act, Indian Residential Schools and Hospitals, the current child welfare and criminal justice systems, ongoing treaty violations) have disrupted the wellbeing, rights, and self-determination of Indigenous Peoples. Concomitantly, these and other forms of structural violence have created and sustained the conditions in which Indigenous Peoples in Canada face barriers to determinants of good health, thereby contributing to significantly higher morbidity and mortality rates among Indigenous Peoples relative to non-Indigenous people [41, 42]. Within this context, systemic racism – including in healthcare settings – has exacerbated the harms experienced by Indigenous Peoples who use/inject drugs, as evidenced by inequitable rates of HCV, HIV, overdoses, and criminalization related to drug offences [26, 43–46]. As one example, recent public health surveillance data from the Canadian provinces of Saskatchewan and Ontario estimated that HCV rates are 6–11 times higher among First Nations Peoples relative to non-Indigenous people [26, 47, 48]. Further still, recent research has demonstrated that Indigenous Peoples in Canada are up to 50 % less likely than non-Indigenous people to be able to access and begin HCV treatment [49, 50], and three times more likely to die without ever having accessed HCV care [26, 51, 52]. It is in this context of ongoing harms associated with systemic racism and related inequities that the current study is situated.

### Sampling and recruitment procedures

Drawing on a stratified purposive sampling strategy [53], we led targeted recruitment of specific participant subgroups (i.e., stratified by gender identity, HIV serostatus, and stage of HCV treatment) from three large prospective cohort studies in Metro Vancouver: the Vancouver Injection Drug Users Study (VIDUS), the AIDS Care Cohort to Evaluate access to Survival Services (ACCESS) study, and the Preservation of Sustained Virologic Response (Per-SVR) study. As described in detail elsewhere [54], VIDUS and ACCESS are open community-recruited prospective cohort studies that, since 1996 and 2005, respectively, have conducted research (e.g., through baseline and semi-annual interviewer-administered questionnaires, testing for HCV and other blood-borne infections, and clinical monitoring) with HIV-negative (VIDUS) and HIV-positive (ACCESS) people who use/inject drugs. Similarly, initiated in 2017, the Per-SVR study [55] is a prospective longitudinal cohort of people with lived experience of HCV and who have completed or who are currently undergoing treatment with DAAs.

VIDUS, ACCESS, and Per-SVR research staff identified prospective participants by querying their respective cohort study databases. During baseline and follow-up visits for their respective studies, research staff informed prospective participants about the current qualitative study. To be eligible for inclusion in this study, participants were required to live within Metro Vancouver, be 19 years of age or older, be fluent in English, self-identify as a person who injects drugs, have lived experience with HCV, and indicate that they were either (1) considering accessing DAA treatments, (2) presently undergoing DAA treatment, or (3) had recently completed DAA treatment. After participants contacted our research team, we provided additional study information, confirmed eligibility, and scheduled interviews. Participants provided written informed consent prior to data collection activities and were remunerated with a CDN $30 honorarium. Ethics approval was obtained from the University of British Columbia Behavioural Research Ethics Board (#H16-02943).

### Data collection

Between January and June of 2018, our study’s research coordinator (co-author PH) and principal investigator (co-author RK) conducted 56 in-depth, semi-structured interviews that lasted 30–60 min. We held interviews at our research offices in Vancouver’s Downtown Eastside. Our critical theoretical and axiological positioning informed the design of our interview guide, which sought to elicit comprehensive discussions about participants’ perceptions and experiences with DAA treatments, HCV care, and health and healthcare access more generally. Our interview questions related generally to how participants had become aware of and informed about DAA treatments. In addition, we asked participants to describe the circumstances and contexts in which they had accessed (or had not been able to access) DAA treatments. At this point in time, we prompted participants to elaborate on how various individual and relational (e.g., provider-patient dynamics; apprehensions related to previous experiences with Interferon-based therapies, as well as concerns about potential side effects) influenced their experiences and perspectives related to DAA therapies. In addition, we encouraged participants to discuss how broader contextual features of their lives influenced their experiences with HCV and wellbeing more generally; in doing so, we sought to elicit discussion of how socio-structural factors (e.g., features of healthcare delivery systems, peer and community supports, treatment eligibility criteria, marginalization, stigma) influence opportunities to access, adhere to, and complete DAA regimens. Participants also filled out an 8-item socio-demographic questionnaire, which included items related to age, ethnicity, HIV serostatus, HCV treatment status, and sexual and gender identity.

### Data analysis

Data collection and preliminary analysis were conducted iteratively, with later interviews helping to identify gaps in our sample and inform subsequent data collection. This iterative process also informed our assessments of whether we had collected sufficient data to comprehensively address our study objective, as well as our eventual decision to cease recruitment. Interviews were audio-recorded, transcribed verbatim, accuracy checked, anonymized, and securely and digitally stored with identifying details removed. We uploaded the interview data to NVivo 12 software, which we used to manage the analysis. At early stages of the analysis, co-author PH, the lead author (TG), and one non-author research assistant (NT) began by reading and re-reading the interview transcripts to familiarize themselves with the data. PH and NT inductively organized the data into patterns, which were then assigned substantive open codes (e.g., related to: background participant information, experiences living with HCV, experiences and perceptions of DAAs, barriers and facilitators associated with DAA treatment access and uptake) [35]. Each transcript was then independently coded by either PH or NT, using the data-driven coding schematic. With consultation from senior co-authors RK, HB, and AJB, TG then employed axial coding [34, 35] to organize initial codes into “trees” that represented groups of related concepts and categories, which provided a foundational schematic for the analysis. In doing so, we used constant comparative techniques [34] to further distil and contextualize emerging themes.

Throughout data analysis, we explored each key theme more fully by engaging with analytical questions that stemmed from our equity-oriented theoretical perspectives [31–33], including: (i) How do perceptions about HCV and its treatments (i.e., both historical and current) shape PWID’s attitudes and experiences with DAAs? (ii) What key considerations and socio-contextual factors influence opportunities to access and complete DAA treatment? (iii) Under what conditions and in which contexts is equitable access to DAAs and wellbeing more generally for PWID realized? As the analysis proceeded, we addressed discrepancies between emergent themes through debriefing processes at team meetings. In addition, the lead author employed a series of inductive approaches (e.g., returning to the data for nuance and context, iteratively contrasting emerging themes against what is already documented in related empirical and theoretical literature bases) to construct and refine central themes, which we present below.

## Results

A total of 56 PWID with lived experience of HCV were included in this analysis. Table 1 provides an overview of the socio-demographic characteristics of this sample. In addition, although not explicitly asked in our socio-demographic questionnaire, our interviews surfaced that many participants had experienced – and, in most cases, were still experiencing – significant socio-economic hardship, including living on very low incomes and in inadequate housing situations (e.g., couch surfing, shelters, outside). The social context of participants’ lives is surfaced throughout the analysis below, where we offer the findings in three thematic sections: (i) life with HCV, (ii) experiences with and perceptions of evolving HCV treatments, and (iii) substance use and the uptake of DAA treatments. Each participant quotation is accompanied by a brief description of the participant’s socio-demographic profile and a researcher-assigned numerical identifier.

**Table 1:** Characteristics of participants

Participants	56
**Age (average, range)**	**49** (31-66) Years
Ethnocultural identity	
*First Nations*	**28** (50%)
*Métis*	**3** (5.4%)
*Black*	**1** (1.8%)
*White*	**19** (33.9%)
*Declined to answer*	**5** (8.9%)
HCV treatment status	
*Pre-treatment*^*1*^	**25** (44.6%)
*Peri-treatment*	**12** (21.4%)
*Post-treatment*	**19** (33.9%)
HIV serostatus	
*Positive*	**27** (48.2%)
*Negative*	**29** (51.8%)
Sexual identity	
*Heterosexual/straight*	**42** (75%)
*Bisexual/bicurious*	**4** (7.1%)
*Lesbian*	**1** (1.8%)
*Gay*	**1** (1.8%)
*Two-Spirit*^*2*^	**2** (3.6%)
*Other*^*3*^	**2** (3.6%)
*Declined to answer*	**4** (7.1%)
Gender identity	
*Man*^*4*^	**29** (51.8%)
*Woman*^*5*^	**26** (46.4%)
*Two-Spirit*^*2*^	**1** (1.8%)

^1^This category includes one participant whose completed DAA treatment regimen did not result in cure, one participant who prematurely ceased DAA treatment due to adverse side effects, and one participant who re-acquired HCV after being cured with Interferon-based therapies several years prior. All of these participants expressed intent to (re)access DAA treatment.

^2^“Two-Spirit” is an umbrella term intended to encapsulate a range of Indigenous gender diverse and non-normative sexual orientations [56]. There is no singular definition of this term, as its use varies across and within Indigenous Peoples and communities. Two participants in this study described their sexual identities as Two-Spirit, whereas another participant used this term to refer to their gender identity.

^3^In this category, one participant identified as transgender and another participant identified as androgynous. Although we associate these terms with gender identity and expression, this table presents the sexual identities indicated by participants themselves.

^4^All men who participated in this study identified as cisgender.

^5^One woman who participated in this study identified as transgender, whereas the remaining women identified as cisgender.

### Life with HCV: “We’re not really given all the information"

As the interviews began, participants reported having variable and sometimes limited amounts of clinical information related to HCV and its treatments. Among participants who had not yet accessed DAA treatment, in particular, several described experiences in which they had not been adequately informed by their healthcare providers about the meaning and potential impact of HCV (e.g., symptoms, transmissibility, prognosis, treatment options). Indeed, some of these participants even indicated that, through the interview questions and prompts regarding DAA treatments for the present study, they were being informed of DAAs for the very first time. Here, participants also described how the prevalent and often asymptomatic nature of HCV within their communities had led to HCV care being conventionalized and deprioritized by some healthcare providers. These participants further postulated that this “downplaying” of HCV had inadvertently affected the amount of HCV-related information they had been given and the extent to which they had been engaged by their healthcare providers in HCV care. Some participants indicated that HCV care continues to be deprioritized in today’s healthcare context, relative to other health concerns (e.g., overdose, HIV). One 53-year-old woman, who had not yet been able to access HCV treatment, described this critical information gap when outlining her experience of being diagnosed with HCV by her family physician:

*Hep C is the least of the dangers [compared to other illnesses], but it doesn’t mean it’s not dangerous. And we’re not really given all the information about what organ it [HCV] hurts, what exemplifies it, or what could help on a daily basis to avoid it. Like, is it a growth, is it a, you know, a virus, like a liquid, or is it hardening or, you know, I don’t know any of those things (Participant_17).*

Conversely, some participants described healthcare interactions in which they had been “overloaded” with information related to HCV and other aspects of their health, including, in particular, substance use and HIV. These participants described instances in which they had been unsatisfactorily supported by their healthcare providers and how, within this context, the shock of being diagnosed with HCV – and, in many cases, also HIV – had caused them to “close down” and not retain important information related to their illness and/or potential treatment options. More generally, several participants pointed to the ways in which previous and ongoing negative experiences within clinical encounters could impact their subsequent experiences and trajectories of care. For example, participants described highly dehumanizing clinical encounters (i.e., that lacked respect, empathy, and recognition of client choice) with healthcare providers, which they tended to associate with their ongoing mistrust of some healthcare providers. As such, participants emphasized that negative experiences with healthcare providers were strongly tied to a deep hesitancy they have around seeking follow-up HCV care, including DAA treatment. As one prominent example, one 55-year-old woman undergoing HCV treatment recounted the context in which, while in her 30s, she and her newborn son had both been tested for HCV and HIV:

*I found out about my hepatitis C when I found out I had HIV. [. .] I went and seen him [the physician], and he tested my son, he tested me. He said, “Come back in two weeks.” And when that two weeks came by, I went and seen him [again]. He goes, “Well, I’ve got some good news and I’ve got some bad news.” And I said, “What’s that?”. He says, “Well, first of all, you have hep C.” I said, “Okay.” And he goes, “And your son’s gonna live, but you, you’re gonna die.” I said, “What?” [laughs]. He goes, “Because you have HIV.” I said, “Okay.” And then, when he said I was gonna die, I just closed right down. . I didn’t hear a word what he said (Participant_10).*

As participants’ stories further unfolded, a subset described how, despite having lived with HCV for years or even for decades, they had largely been asymptomatic of HCV, and therefore had tended to de-prioritize seeking HCV-related information and/or treatment. In describing how she had lived with HCV for more than 20 years, one person reflected:

*“I wasn’t really worried about it [HCV] because I was young and still healthy” (Participant_19; 46-year-old woman, also living with HIV, completed HCV treatment).*

Some of these participants described how they had nonetheless begun treatment after being encouraged to do so by their healthcare providers during hospitalization or while accessing community-based healthcare for other co-morbidities. Characterized within these descriptions was a sense that decentralized, primary care-oriented HCV care had become increasingly commonplace in their local healthcare context. Alongside these healthcare system adaptations, many participants also indicated that their experiences of aging, the intensifying burden of late-onset and chronic HCV symptoms (e.g., fatigue, insomnia, depression, pain, jaundice), and, in many cases, the increasing toll and stress of living on a low income and/or in substandard housing had shaped a set of conditions in which they felt they needed to access HCV treatment. Here, one 45-year-old man, who had been living with HCV for more than 20 years prior and who had not yet been able to access treatment, described the subtle but gradual and regressive nature of HCV disease progression, which reinforced his present motivation to seek medical attention:

*I just kind of [thought], like, “Oh, I’m young, you know. I’ll ignore it [HCV]. I’ll be alright and I’ll fight it off. I’ll be alright, you know. Now, I’m kind of wanting to [learn more about it], because I’m not getting frigging younger here, right? (Participant_04).*

In summary, participants described how their healthcare interactions and the timing and impact of their symptoms while living with HCV impacted their knowledge, motivations, and experiences with HCV care access, including DAAs. In considering participants’ portrayals of life with HCV, we continue the analysis below by explicitly identifying participants’ perceptions and experiences related to HCV treatments.

### Experiences with and perceptions of evolving HCV treatments: “The new one is way better than the old one”

The majority of participants described how, within the last 1–2 years (i.e., contemporaneously with the introduction of universal access to DAAs in BC, in 2018), they had become aware of DAA treatments through discussions with members of their peer and healthcare networks. Yet, several participants described continued uncertainty as to whether or not they were eligible for DAA treatments – particularly, if they had previously been denied Interferon- and/or DAA-based HCV treatments. Here, several participants also described a sense of ambiguity related to where, when, and how they could access DAA treatment. Amidst these descriptions, many participants’ stories chronicled the challenge of identifying and accessing healthcare services and providers with whom they could potentially begin DAA treatment regimens. For example, the above participant further explained:

*I don’t know where to get it [DAAs], or if there’s any out there, or if we’re eligible for it. There’s not too much information about it, it seems like, you know? I want to get better, but there’s not too many places (Participant_04).*

Similarly, several participants – namely, those who themselves had undergone Interferon-based treatments but who continued to be living with HCV – expressed ongoing apprehension about accessing DAA therapies, as they anticipated that the side effects would not be tolerable. During these discussions, it became apparent that participants had not received accurate information related to DAAs, as some were unaware that many of the side effects associated with Interferon-based treatments did not apply to DAA regimens. One 41-year-old woman described her deliberation about whether or not to access treatment with DAAs, which, at the time of the interview, she was about to begin:

*“I was thinking about the side effects. Yeah, what with… like, I want to know what… if I took it [DAAs], what’s the side effects is, I guess. Yeah. I wouldn’t know, because I don’t know what kind of side effects it would affect on me, right? About taking the [DAA] pill” (Participant_03).*

In some interviews, participants described a sense of mistrust and skepticism toward the interests and motivations of HCV-related public-health and pharmaceutical-research officials. By association, these participants expressed significant caution and hesitancy regarding the safety of DAA treatments. Among these participants, some expressed skepticism that they might be treated as “guinea pigs” for experimental HCV treatments, which, in some cases, contributed to hesitancy to “take up” DAAs. For instance, while being prompted about DAAs by the interviewer, one woman, who was receiving HCV treatment and who opted not to disclose her sociodemographic data, described:

*[DAAs] cost so much money. Now, why is it 700 dollars a pill now? This is what I was trying to find out, too: is this to cover the cost of the research before they can make the generic pills? Or, how come it costs so much money right now? [. .] Was it tested on animals? [. .] I don’t want to be a guinea pig. (Participant_52).*

Nonetheless, amidst descriptions of learning about DAAs, several participants expressed excitement and interest in novel HCV treatment regimens, which, as participants further described, had often been presented to them (i.e., by peers, healthcare providers, and online resources) as more tolerable and more effective medications than Interferon-based therapies. Often, participants contrasted the perceived opportunities presented by DAA treatment regimens with their previous experiences with and perceptions of Interferon-based therapies. In doing so, participants frequently characterized negative attributes of Interferon-based therapies, including their adverse side effects, prolonged treatment durations, and relative ineffectiveness when compared to DAA treatments. For instance, one 47-year-old man, who had not yet been able to access HCV treatment, described how:

*I know that there was a lot of side effects to it [Interferon-based treatments]. That’s what I heard about it. But the new one [DAAs] just kind of got me right off of it [referring to symptoms of low energy]. A lot of people are finding themselves getting treated of it [HCV], get cured of it, like, real quick. So yeah, the new one is way better than the old one, as far as from what I hear (Participant_07).*

Concerns related to treatment side effects were further described by another participant who had not yet been able to access HCV treatment:

*I was really scared because my friend did the [Interferon-based] treatment and he did not look the same. I thought he was going to die. [. .] That scared me, and I said I wasn’t going to do it [treatment], until now I heard about the [DAA] treatments now, that they’re a little bit… you don’t get no side effects. So, I’m really looking forward to that, kind of thing (Participant _048; 42-year-old Two-Spirit person).*

In considering participants’ accounts of their histories with HCV and Interferon-based treatments, the data highlighted how the implementation of DAAs represents both a pivotal opportunity and a significant period of adjustment and uncertainty (e.g., related to treatment eligibility, side effects, access, and potential outcomes) for PWID living with HCV. Specifically, perceptions of DAA treatments and uptake of DAAs are deeply shaped by an array of experiential factors, including individual, interpersonal (e.g., peer influences), and community experiences with HCV and its treatments.

### Substance use and the uptake of DAA treatments: “You don’t have to quit using now, but you can’t miss any doses once you start treatment”

Almost all participants described the ways in which their substance use, including alcohol, and related engagement with primary care and harm reduction services could be both a potential barrier and/or facilitator to equitable DAA treatment access. For example, in recounting their experiences across both Interferon- and DAA-based treatment eras, several participants described instances in which their substance use had been – and, in some cases, continues to be – characterized by healthcare providers as a contraindication to HCV treatment eligibility, despite this not being a policy-mandated contraindication. Indeed, the majority of participants described experiences wherein their physicians had either explicitly withheld HCV treatment, or recommended that participants stop or greatly reduce their substance use prior to accessing treatment. This denial of access to HCV treatments was described by one participant:

*He [the physician] just wanted me to quit drinking [before I could start treatment], that’s all. And I could see his point. Yeah, but to force me to quit drinking and then say you’ll help me, that’s not right. I was living in squalor. I was couch surfing and everything and I said I want to get my own place, and he wouldn’t help me. [. .] Then I moved to [name of another physician], and he got me right on it [DAA treatment], and then he cured me. So, big difference of doctors, isn’t it? (Participant_50; 52-year-old woman, completed HCV treatment).*

As illustrated above, several participants described how they responded to healthcare provider gatekeeping of DAAs by seeking out more person-centered, equity-oriented, and power-balanced sources of HCV care. Participants’ accounts of navigating HCV services therefore highlighted their resiliency and determination in finding service providers who did not reproduce systemic barriers to safe, nonjudgmental, and high-quality healthcare. For example, participants described how they valued healthcare providers whose approaches to HCV care were supportive and grounded in harm reduction, as opposed to abstinence-based approaches. One 47-year-old man, who had not yet been able to access HCV treatment, explained:

*[When I was diagnosed with HCV, six years ago], they [the healthcare providers] told me that it is treatable, right? But you have to be willing to stop doing this and stop doing that. I’m like, “I’m not willing to stop anything.” Like, using heroin and crack and coke and all of that B.S [bullshit]. But now that I cut myself down off of everything else, and I just stick to one dope now, which is heroin, [my current physician] said, “You don’t have to quit using now. You could still take your pill while you’re doing whatever it is you’re doing. But you can’t miss any doses once you start,” right? (Participant_07).*

Several participants postulated that being able to have transparent and supportive discussions about their substance use facilitated open communication and the development of individualized HCV treatment plans. Participants further described how, in collaboration with their harm reduction-oriented healthcare providers. they had planned and implemented strategies for making HCV treatment more accessible and thereby more effective. These plans frequently included the integration of DAA treatments with other substance use-related services (e.g., OAT, HIV care, outreach and in-reach harm reduction services). Here, a subset of participants described how their inclination to integrate DAAs with existing services stemmed from their concerns that they might otherwise forget to take their doses (and thereby risk making the treatments ineffective); yet, the majority described how integrating services was simply a matter of convenience. One participant described how DAAs were incorporated into her daily routine of acquiring OAT (in her case, methadone) from her pharmacist:

*Well, you just give it [DAAs] to them at the pharmacy then. Mine was taken every day at the pharmacy. It’s what I asked of them [my physician], “When I go get my methadone, just give it to me with that.” (Participant_24; 54-year-old woman, completed HCV treatment).*

A subset of participants described how their treatment plans were made even more comprehensive through the involvement of multiple supports and services, including peers, partners, family, and housing and outreach workers. This support network was described as a sort of “safety net,” who, if needed, could remind participants to take their DAA doses. Similarly, some participants described how logistical and organizational features of support services (e.g., extended and weekend hours of operation, the potential to carry take-home doses of DAAs from pharmacies) could serve to promote treatment accessibility and adherence. At times when they were holistically supported, participants described feeling confident and optimistic about their experiences with DAAs:

*I never forget [to take my DAAs], now. [. .] I’m very vigilant now. I get up in the morning times, so I’m vigil[ant], so I know what I’m doing. [. .] And, plus, the pharmacy’s aware of my situation, and knows that they have to phone me at a certain time to remind me that I have to come in. And, I’m grateful for that. Plus, I have the people at my apartment building [i.e., outreach workers] – they’re aware of my situation now, too. So, they come and do an eight o’clock wake-up call with me, to remind me to take my medication. So, I’ve got it down (Participant_05; 53-year-old woman, undergoing HCV treatment).*

Across these findings, participants described how various features of the healthcare system and provider-patient interactions could reduce barriers and thereby promote opportunities for equitable DAA access and uptake. In particular, participants described the diverging ways in which their healthcare providers had framed their substance use as either a barrier or, through harm reduction-oriented approaches, a potential avenue for engaging participants in HCV care, including DAA treatments.

## Discussion

The introduction of universal DAA coverage in many settings, including BC, Canada, has transformed the HCV treatment landscape and has expanded opportunities to treat priority populations, including PWID. Yet, as these findings highlight, significant social and structural barriers to DAA treatments for PWID remain heretofore unaddressed. Drawing from participants’ extensive experiences with HCV and related healthcare system engagement, findings from this study underscore how health and healthcare practices and policies, such as the deprioritizing of HCV (e.g., due to being asymptomatic, healthcare provider gatekeeping) and catalysts to care (e.g., symptom onset and burden, treatment for co-morbidities), shaped experiences with and access to DAAs. More broadly, participants described how experiences with evolving HCV treatments (e.g. [in]eligibility, side effects, skepticism) and overarching approaches to care (e.g., abstinence-based, harm reduction-oriented, integrated) influenced motivations and opportunities related to DAA treatment access.

Findings from this study indicate that HCV-related care trajectories and clinical encounters are often fraught with uncertainty and misinformation. These findings identified how poor healthcare-provider engagement and support can lead to gaps in people’s knowledge about HCV, particularly with regard to potential consequences (e.g., symptoms, impacts on quality of life), as well as its treatment. Furthermore, these data underscore the extent to which stigmatizing and dehumanizing healthcare encounters, characterized by a lack empathy and respect, also prevented opportunities for PWID to acquire HCV-related information. The pronounced provider-client power imbalances as the context within which information gaps are occurring represent significant deterrents to the delivery of good care, as HCV illness- and treatment-related knowledge deficits are key barriers to DAA treatment uptake [25, 57]. To strengthen access to equitable healthcare information and treatment, it has been extensively argued that clinicians must continuously attend to power differentials and meaninfully engage clients as active participatns in care [58, 59]. Findings from this study indicate that clinicians involved in HCV care must adopt equity-oriented and history-informed approaches that recognize and address the common concerns PWID may have related to HCV treatments (e.g., previous adverse experiences with Interferon-based therapies, concerns about DAA treatment side effects, confusion related to treatment eligibility). Unfortunately, however, previous research has indicated that specialist and primary care providers often feel they are inadequately trained to provide care that aligns with equity-oriented approaches, and are therefore insufficiently prepared to provide HCV and substance use-related care [60, 61]. To comprehensively address this provider- and patient-level knowledge gap, we call for expanded advocacy and educational efforts to promote clinician capacity to provide equitable and effective care to PWID living with HCV. Indeed, these findings identify the extent to which healthcare providers need to better mitigate unequal provider-client power relations and the corresponding impacts on health and healthcare access for PWID.

Relatedly, these findings provide a critical glimpse into how structural barriers, including substance use stigma, restrict opportunities for DAA treatment uptake among PWID. For example, participants in the current study generally described how HCV care had tended to be deprioritized by healthcare providers on the basis of active substance use. In addition, findings from this study surfaced other clinical experiences in which guiding ethical principles of care were not followed (e.g., clinicians coldly framing HCV/HIV as a “death sentence” at the time of diagnosis). In considering these challenges, findings from this study further illustrate that the remaining barriers to HCV care identified herein (e.g., misinformation, gatekeeping) are entwined with stigmatization and mistreatment, which are well-documented determinants of HCV-related and other health and social inequities among PWID [7, 27, 30, 62–65]. These findings are consistent with previous research suggesting that PWID, including those living with HCV, tend to be treated as passive recipients of care, not meaningfully consulted in discussions surrounding their health and wellbeing, and labelled and stigmatized within healthcare settings – and that their living circumstances (e.g., related to: substance use, income, housing) are often framed as contraindications to care, rather than carefully considered within person-centered care strategies/plans [6, 66–69]. Findings from this study therefore underscore the need for healthcare providers – particularly, those with prescriptive authorities for DAAs (e.g., physicians, nurse practitioners) – to treat PWID according to fundamental ethical principles (e.g., compassion, dignity, respect for persons) underpinning clinician codes of ethics for socially just clinical practice (e.g., [70, 71]). Further, these findings highlight the importance of facilitating access to HCV care while taking seriously the need for larger scale system-level and structural changes and “upstream” policy responses (e.g., safe housing for everyone).

Acknowledging the colonial context in BC, the perspectives of Indigenous Peoples in this study sample, and the harmful clinical encounters described herein, findings from this study also align with a growing body of Canadian and international empirical evidence highlighting how HCV-affected Indigenous Peoples are distinctly and inequitably mistreated within healthcare settings [26, 72–74]. For Indigenous Peoples, including those who use substances and who face substance use stigma, historical and ongoing contexts of systemic racism and colonialism are known to create barriers to safe, effective, and timely healthcare [58, 75]. To mitigate ongoing health inequities, including those stemming from structural barriers to clinical care, HCV treatment providers must take meaningful action to create and maintain relationships that are safe and trauma- and history-informed, and that promote equitable access to care, information, and treatment for Indigenous and other PWID living with HCV. Here, there is a critical need for culturally safe approaches that foreground social justice goals in HCV care provision and that include clinician “critical self-refection of biases, acknowledgement of power imbalances, and conviction to uphold Indigenous [and non-Indigenous] patient self-determination at every step of the HCV cascade of care” (26p60), along with structural interventions to redress inequities related to HCV treatment, care and outcomes.

To further optimize DAA treatment experiences among PWID, these findings also indicate the need for system-level changes, including the provision of low-barrier, integrated, and peer-led services. For example, participants emphasized how their relationships with harm reduction-oriented healthcare providers facilitated opportunities to develop comprehensive HCV treatment plans, which included linking DAAs with existing services that many PWID already access – the feasibility and effectiveness of which has documented elsewhere, such as in the contexts of HIV care [76, 77] and OAT provision [18, 78–82]. In addition, participants described how efforts from community members and housing-support workers to facilitate and uphold DAA treatments plans (e.g., through “check-ins” and reminders to take medications) further contributed to the consistent uptake of DAA treatments. The importance of peer and social supports was further echoed in participants’ descriptions of community members as trusted sources of knowledge who essentially vouched for DAAs, such as by alleviating potential concerns (e.g., related to side effects and eligibility) and by substantiating the safety and effectiveness of novel treatments. These findings align with recent research underscoring the influence of peers (i.e., other PWID with lived experience of HCV) on health seeking behaviours and the spread of health information, particularly in regard to DAA treatments [20, 83–85]. To further promote linkages to HCV care and the equitable scale-up of DAAs among PWID, additional public health efforts to implement and optimize peer-driven and network-based interventions are warranted.

This study has several strengths and limitations. The large and diverse sample of PWID who have lived experience with HCV yielded highly-contextualized descriptions of HCV care across both Interferon- and DAA-based treatment eras. Nevertheless, we acknowledge that the perspectives of other stakeholders in HCV care (e.g., peers, family members, clinicians, policymakers) were beyond the scope of this research. In addition, limitations in the study design and the specificity of the research questions hindered our ability to meaningfully investigate how perceptions and experiences with DAAs vary across and within subpopulations of PWID (e.g., across axes of ethnocultural identity, socio-economic status, sexuality, gender identity, and HIV serostatus; across participants’ “types” and contexts of substance use); we were therefore unable to delineate significant differences across these population sub-groups. Further research is needed to explicate ways in which access to DAA treatment is embedded within intersecting socio-structural contexts, and how these contexts may differentially impact PWID based on various aspects of social location. Similarly, although participants often contrasted their experiences with DAAs to their past experiences during the Interferon-based HCV treatment era, we had not explicitly sought to investigate changes in these experiences over time. Historical and/or longitudinal analyses of evolving HCV care landscapes could offer additional insights into the ways in which policy and programmatic transformations directly influence the health experiences and outcomes of PWID with lived experience of HCV.

## Conclusions

The introduction of novel DAA treatments and the subsequent removal of regulatory barriers to access these medications in many settings, including BC, Canada, has renewed optimism for expanding HCV treatment efforts. To further promote the equitable scale-up of DAAs among PWID, comprehensive approaches that account for the socio-structural and historical factors that have influenced HCV-related health and healthcare access for this population are needed. Findings from this study underscore several healthcare and service delivery transformations that are required to meaningfully facilitate PWID’s access to DAA treatments, including the scale-up of integrated services and peer- and community-based interventions and supports, alongside the championing of equity-oriented clinician approaches to care that are attentive to power differentials and the social contexts of people’s lives, culturally safe and non-stigmatizing, and grounded in harm reduction.

## Data Availability

The data analyzed during the current study are not publicly available because they contain information that could compromise research participant privacy and consent, but are available from the corresponding author on reasonable request.

## Abbreviations

BC: British Columbia
DAA: Direct-acting antiviral
HCV: Hepatitis C virus
HIV: Human immunodeficiency virus
PWID: People who inject drugs
OAT: Opioid agonist therapy

## Footnotes

1 The term “Indigenous” refers to First Peoples internationally. We use this term to broadly refer to the diverse groups of First Nations, Métis, Inuit, and other Indigenous Peoples living in Canada.
